# Insulin signaling and its application

**DOI:** 10.3389/fendo.2023.1226655

**Published:** 2023-08-17

**Authors:** Thi Kim Chung Le, Xuan Dat Dao, Dang Vung Nguyen, Duc Huy Luu, Thi Minh Hanh Bui, Thi Huong Le, Huu Thang Nguyen, Tran Ngoan Le, Toshio Hosaka, Thi Thu Thao Nguyen

**Affiliations:** ^1^ School of Preventive Medicine and Public Health, Hanoi Medical University, Hanoi, Vietnam; ^2^ Department of Biopharmaceuticals, Institute of Chemistry, Vietnam Academy of Science and Technology, Hanoi, Vietnam; ^3^ Department of Food and Nutritional Sciences, University of Shizuoka, Shizuoka, Japan

**Keywords:** insulin signaling, insulin resistance, application, insulin signal pathway, receptor, post-receptor signal transduction

## Abstract

The discovery of insulin in 1921 introduced a new branch of research into insulin activity and insulin resistance. Many discoveries in this field have been applied to diagnosing and treating diseases related to insulin resistance. In this mini-review, the authors attempt to synthesize the updated discoveries to unravel the related mechanisms and inform the development of novel applications. Firstly, we depict the insulin signaling pathway to explain the physiology of insulin action starting at the receptor sites of insulin and downstream the signaling of the insulin signaling pathway. Based on this, the next part will analyze the mechanisms of insulin resistance with two major provenances: the defects caused by receptors and the defects due to extra-receptor causes, but in this study, we focus on post-receptor causes. Finally, we discuss the recent applications including the diseases related to insulin resistance (obesity, cardiovascular disease, Alzheimer’s disease, and cancer) and the potential treatment of those based on insulin resistance mechanisms.

## 1 Introduction

Insulin signaling beginning at its receptors produces multidirectional impacts on the metabolism, survival, and proliferation of targeted cells. Insulin transduces its effects by insulin receptors that signal through many pathways including participation in protein and lipid phosphorylation, management of trafficking procedures, management of a system of enzymes, and control of a system of transcriptional factors. Numerous studies have indicated that insulin resistance is related to many diseases: type 2 diabetes, obesity, cardiovascular disease, Alzheimer’s disease, and cancer, which are the medical challenges in the first century of the new millennium (1–3). Therefore, it is necessary to update the physiology of insulin signaling and the disordered physiological processes associated with insulin resistance, henceforth proposing more effective therapies for treating diseases related to insulin resistance.

## 2 Insulin signaling pathway

**Figure 1:**
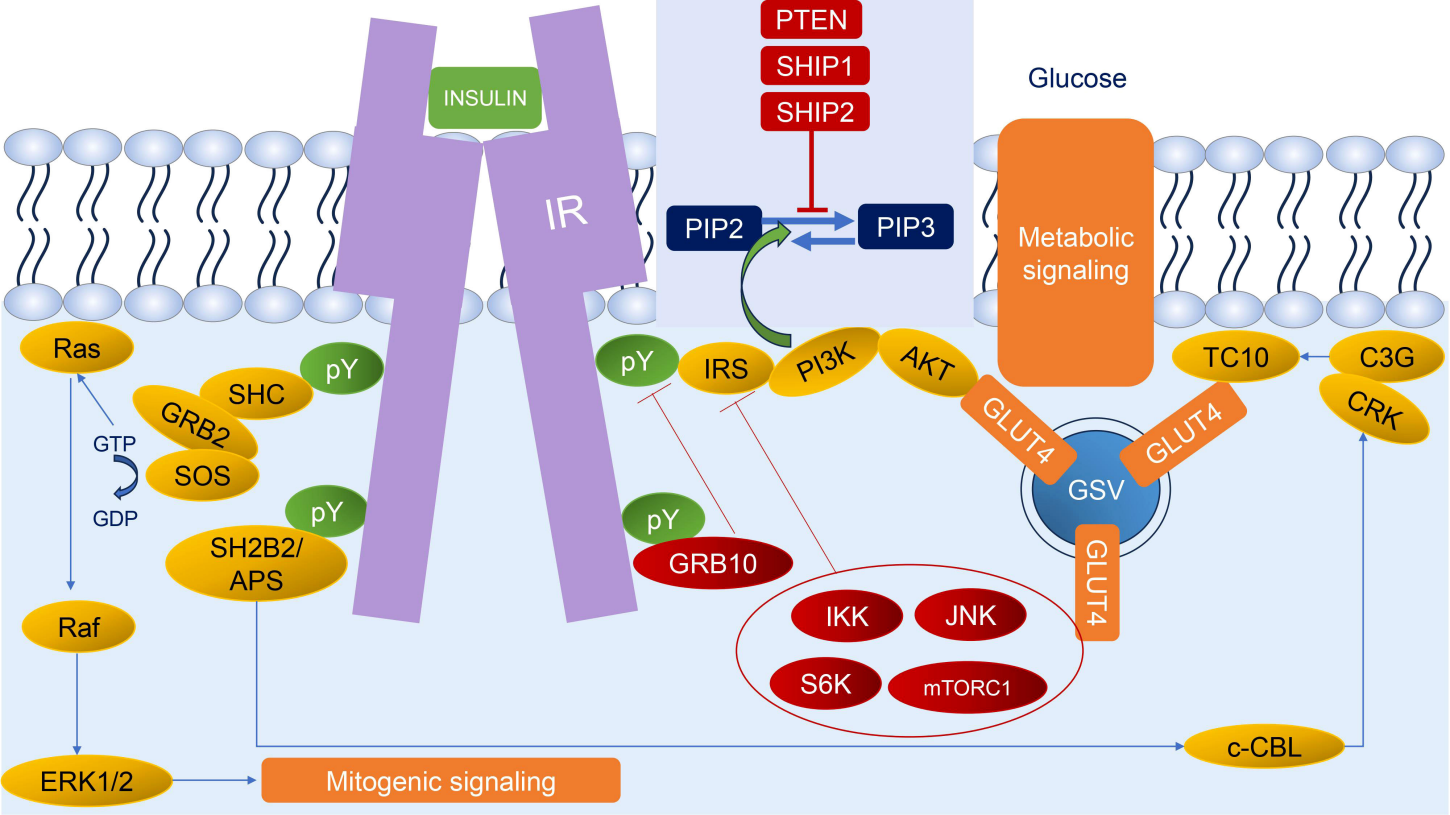
Insulin signaling. Insulin binds to IR leading to autophosphorylation of IR; thus, IR could recruit diverse substrates. The two main responses of insulin signaling are mitogenic signaling (begin with SHC and GRB2 through the ERK1/2 pathway) and metabolic signaling (begin with IRS through the AKT pathway and SH2B2/APS through CRK/TC10 pathway). The regulation of insulin signaling could be characterized by negative feedback mechanisms, such as stabilization and recruitment of GRB10 to the IR; activation of lipid phosphatases (PTEN, SHIP1, and SHIP2) that dephosphorylate PIP3; and activation of several stress kinases (IKK, JNK, S6K, and mTORC1) to phosphorylate and inhibit IR and IRS. Green circles and arrows represent activating events; red circles and lines represent inhibitory events. pY: phosphorylated tyrosine residue.

The insulin signaling pathway is an intracellular signaling pathway that is responsible for the metabolism of the body, especially in metabolism, growth, and survival. The process of the insulin signaling pathway comprises several steps, wherein the initial stage is the participation of insulin and insulin-like growth factors (IGF) which bind to insulin and IGF receptors. The second step is binding an insulin receptor (IR) to its direct substrates including Growth factor receptor-bound protein 2 (GRB2), Src homology 2 domain-containing (SHC), insulin receptor substrate (IRS), SH2B adapter protein 2/adapter protein with a PH and SH2 domain (SH2B2/APS), and Growth factor receptor-bound protein 10 (GRB10). This binding could exert several cellular signaling pathways for mitogenesis and metabolism. Our review will briefly unravel every step of the insulin signaling pathway (2) (**Figure 1**).

Insulin is classified in the peptide hormone group that is secreted from beta cells in the islet of Langerhans of the pancreas. In mammals, apart from insulin, IGF-1 and IGF-2 are also synthesized by the insulin gene family. Insulin could bind to the specific receptors on target cells and exert the metabolic response. Similarly, IGF-1 and IGF-2 would trigger the mitogenic response, and promote proliferation and differentiation of cells (4, 5). Nevertheless, these functional distinctions are blurry due to the high similarity between the insulin and IGF receptors and are common in many downstream effectors (3, 6). Therefore, there is a relationship between hyperinsulinemia and neurodegeneration, cardiovascular disease, and several cancers (7–9).

IR is a heterotetrameric receptor tyrosine kinase that was formed by four subunits composed of two extracellular α subunits and two transmembrane β subunits. Owing to this structure, IR has two isoforms, A and B, and there are several differences between them. IR-A, generated by the splicing out of exon 11 that leads to the absence of 12 amino acids at the carboxyl terminus of the α subunit, is highly expressed during fetal life and has more potency in metabolism, promoting glycogenesis compared to IR-B. IR-B is differentiated by alternative splicing and includes an additional 12-amino acid sequence encoded by exon 11 at the C-terminal of the α subunit; the corresponding sequence in type 1 IGF is vacant. The presence of IR-B in the liver, muscle, and white adipocyte tissue is more predominant than IR-A due to the high affinity of IR-B compared to IR-A (6, 10, 11).

Insulin binding to IR creates trans-autophosphorylation into IR, which is an essential event for the recruitment of IR’s direct substrates. These substrates bind to IR by the bindings between the phosphotyrosine-binding (PTB) domain of the substrates and the tyrosine residues of IR. The downstream signaling of IR activation comprises two parts: metabolic and mitogenic signals. The metabolic signals need a lower insulin concentration to be triggered compared to the mitogenic signals (2, 3).

### 2.1 The metabolic response

The metabolic arm of insulin signaling is initiated by IRS and SH2B2/APS (2). Together, these pathways warrant the effective translocation of glucose transporter type 4 (GLUT4) to the membrane by adapting signaling platforms at the cell surface which are composed of lipids, protein kinases, small GTPases, and adaptor proteins. To be specific, GLUT4 is in the GLUT family of transmembrane hexose transporter, which has a high attractiveness to glucose, and the majority of it is expressed in muscle cells and adipocytes (12). The translocation of GLUT4 in response to insulin or exercise results in a ten-fold increase in glucose uptake (13); when insulin is absent, only approximately 5% of the total GLUT4 is found on the membrane (14, 15). GLUT4 trafficking has a key role in the maintenance of glucose homeostasis by preventing gluconeogenesis in the liver and promoting glucose uptake into the muscle and adipose tissues, but it depends on the response to insulin and coordination of insulin signaling (PI3K signaling pathway and APS signaling pathway) in every step of GLUT4 trafficking, including GLUT4 endocytosis, GLUT4 sorting and retention, and exocytosis of GLUT4 storage vesicles (GSVs) (12).

The best-unraveled substrate for the role of IR scaffolds is IRS. The IRS family has six members, wherein IRS1 and IRS2 are claimed to mediate virtually the metabolic responses of IR activation. IRS has NH2-terminal pleckstrin homology (PH) and phosphotyrosine-binding domains (PTB domains) which are responsible for guiding them to activate IR. After binding to IR, the IRS tyrosine residues are phosphorylated which enlist downstream effectors to enhance the insulin response (16). Particularly, phosphorylated IRS attract phosphoinositide-3-kinase (PI3K) heterodimers containing a regulatory subunit with SH2 domain (p85) and a catalytic subunit (p110) that stimulate glucose transport (17, 18). Besides, PI3K catalyzes the reaction, converting phosphatidylinositol-4,5-biphosphate (PIP_2_) to phosphatidylinositol-3,4,5-triphosphate (PIP_3_). On the contrary, phosphatase and tensin homolog deleted on chromosome 10 (PTEN) catalyze the reverse reaction. Interestingly, insulin could block the activity of PTEN by obscure mechanisms, which may be explained by the interactions with phosphatidylinositol 3,4,5-trisphosphate-dependent Rac exchanger 2 (P-REX2) through two joints, i.e., PH domain of P-REX2 binding to the catalytic region of PTEN and the inositol polyphosphate 4-phosphatase domain of P-REX2 interacting with the postsynaptic density-95/Discs large/zona occludens-1-binding domain of PTEN. These interactions request the phosphorylation of the C-terminal of PTEN to discharge the PH domain of P-REX2 from its adjoining diffuse B-cell lymphoma homology domain (19). Therefore, activating PI3K as well as inhibiting PTEN by insulin leads to the accumulation of PIP3. PIP3 then pulls in proteins with PH domains to the plasma membrane including phosphoinositide-dependent kinase 1 (PDK1) and Protein kinase B/AKT. PDK1 phosphorylates AKT in its activation loop after binding to PIP3, and the mechanistic target of rapamycin complex 2 (mTORC2) phosphorylates its hydrophobic motif (20–22). AKT is a bridge protein that connects insulin signaling to downstream effectors of GLUT4 trafficking. It is involved in glucose absorption in the muscle and adipose tissue and increases intracellular glucose production (12, 23). AKT promotes GLUT4 storage vesicle (GSV) exocytosis by phosphorylating and dephosphorylating RAB GAP AS160 (also known as TBC1 domain family member 4, TBC1D4) and the RAL-GTPase-activating protein complex (RAL-GAP complex, RGC), which control small GTPases involved in GSV retention and localization to the cell surface. Particularly, RAB GAP AS160 is necessary for insulin activation of GLUT4 exocytosis. The evidence supporting this process is that the inhibitory-induced mutant of AS160 inhibits insulin activation of exocytosis at a prior step of the fusion of GSVs with the cell membrane. Nevertheless, this mutant does not constrain insulin-induced inhibition of GLUT4 endocytosis. RGC is a complex, including an RGC1 regulatory subunit and an RGC2 catalytic subunit, which directly activates the guanosine triphosphate hydrolysis of RalA, whereas the small GTPase RalA downstream of PI3K plays the role as an essential part of redistribution of GLUT4 by marshalling the exocyst complex for GLUT4 vesicle targeting in adipocytes. Thus, RGC complex regulates the transport machineries responsible for GLUT4 translocation, connecting with PI3K signal (12, 24, 25).

Another pathway of metabolic insulin response is the APS-insulin signaling pathway. Activated IR recruits APS, which contain PH and Src homology 2 (SH2) domains and is so also known as SH2B2, with high affinity. Following that, APS pulls in a complex that includes the proto-oncogene c-CBL and c-CBL-associated protein (CAP) and phosphorylate c-CBL (12, 26). Activated c-CBL recruits CRK, which is a complex with the guanine nucleotide exchange factor (GEF) C3G to the plasma membrane, then C3G activates TC10, a member of the RHO-family of small GTPases (12, 27, 28). GTP-bound TC10 interacts with the exocyst tethering complex, resulting in GSV sites docking at the cell surface. Apart from that, TC10 also binds to CDC42-interacting protein 4 (CIP4), forming a stable complex with Rab GEF GAPex5 and regulating the activity of Rab5 family GTPases involved in GSV retention and delivery (12).

### 2.2 The mitogenic response

The second major response of insulin signaling is mitogenic action. In this pathway, insulin acts as a growth factor although it is a considerably weaker mitogen than its cousins, the IGF, and other relatives such as platelet-derived growth factor (PDGF), vascular endothelial growth factor (VEGF), and EGF. It has been considered to be the most anabolic hormone that governs proliferation and migration, and it also inhibits apoptosis (3, 29–31). The mitogenic action is triggered by the binding between IR and SHC protein, which recruits the GRB2-SOS (son of sevenless) complex. SOS regulates the exchange of GTP for GDP only when Ras is attached to the plasma membrane; consequently, SOS GTP loading activates the Ras protein. Farnesylation of Ras facilitates its translocation to the cell surface, particularly when farnesyltransferase (FTase) catalyzes the attachment of a farnesyl moiety to a cysteine residue of Ras. Simultaneously, with the phosphorylating IR, insulin could activate FTase, thus increasing the farnesylation of Ras. Furthermore, insulin-activating FTase requires an intact IR but not an IGF receptor, implying that this action is driven solely by IR and does not include the interaction of IR and the IGF receptor. Ras protein then activates the cascade from Raf protein to the mitogen-activated protein kinase (MAPK). The activated MAPK, extracellular signal-regulated kinase (ERK1/2, also referred to as p44/p42 MAPK), is a key effector of the insulin mitogenic signal that promotes cell growth, cell division, migration, and apoptosis (3, 30). ERK1/2 activates a pleiotropy of substrates (approximately 200 different substrates have been identified), including cytosolic targets (PLA2, Raf-1, Caspase-9, Bcl-2, etc.) and cytoskeletal targets (MAP2, MAP4, Tau, etc.) which regulate specific activities within certain organelles, such as transcription in the nucleus and mitochondria, and bring components of the ERK1/2 cascade into proper localizations where it phosphorylates specific proteins without remarkable nuclear translocation. Half of the currently identified ERK1/2 substrates are nuclear targets including Elk-1, c-Myc, c-Fos, and HIF-1α, which regulate many stimulated nuclear processes, such as transcription, chromatin remodeling, and nuclear translocation (32, 33). Evidence of these effects of the ERK1/2 pathway was found in a variety of tissues, including the adipose tissue, muscle, and pancreatic beta-cells. ERK-1 signaling has been shown to promote adipogenesis, and ERK-1 knockdown animals had decreased adipose mass (3). Pre-adipocytes have very little IR, but as they move through adipogenesis, their IR levels rise to levels that are greater than those of IGF-1R. In studies using cell culture, high insulin dosages stimulate ERK activation and support the early proliferative stage of adipogenesis. ERK-1 activity decreases when adipogenesis increases and IR expression rises, and metabolic insulin signaling takes over. The IGF-1R stimulation is necessary to activate ERK and causes adipogenesis that is provided *in vivo* by high levels of IGF ligands during embryonic and postnatal growth and development in FIRKO mice. As adipogenesis expands and IR expression rises, ERK-1 activation declines, and metabolic insulin signaling takes over. *In vivo*, high levels of IGF ligands throughout embryonic and postnatal growth and development provide the IGF-1R stimulation required to activate ERK and drive adipogenesis, and FIRKO mice with IR missing in adipose tissue, nonetheless, form fat pads (3).

### 2.3 Regulation of insulin signaling pathway

Insulin signaling is always under tight control due to the fact that any abnormal signal could exert severe perturbations in metabolism and tumorigenesis. Hence, rapidly turning off the insulin signal at various levels is critical. Signaling is attenuated by the activity of several phosphatases, stress kinases, and adaptor proteins (34).

#### 2.3.1 Phosphoprotein phosphatases

Tyrosine phosphatases comprise transmembrane phosphatases, such as Leukocyte common antigen-related (LAR) protein and cytoplasmic protein tyrosine phosphatases, especially PTP1B, that could dephosphorylate the tyrosine residues on IR and IRS, thus reducing their actions (2, 34).

The serine/threonine phosphatases comprise protein phosphatase 1 (PP1) and protein phosphatase 2 (PP2, including PP2A, PP2B, and PP2C). PP1 controls several types of enzymes involved in the metabolism of glucose, including glycogen synthase. In the PP2 family, multiple protein kinases linked to insulin function are regulated by PP2A, i.e., AKT, ERK, PP2B, and PP2C (in particular, the PH domain leucine-rich repeat protein phosphatases PHLPP-1 and -2) could dephosphorylate AKT. Thereby, they could induce insulin resistance (35–38).

#### 2.3.2 Lipid phosphatases

Lipid phosphatases, such as PTEN and SH2 domain-containing inositol 5-phosphatases (SHIP) 1 and 2, could dephosphorylate PIP3, hence antagonizing PI3K signaling in cells (34, 37, 39).

#### 2.3.3 Stress kinases

The increased serine/threonine phosphorylation of IR or IRS that has an inhibitor effect on tyrosine kinase activity has been witnessed in IR in humans, thus inhibiting insulin signaling (34, 40). Multiple kinases including c-Jun amino-terminal kinase (JNK), inhibitor of κB kinase (IKK), traditional and innovative PKCs but also mTORC1, and S6 kinase (S6K) could inhibit insulin signaling by increasing serine/threonine phosphorylation and reducing IRS tyrosine phosphorylation (2, 34).

#### 2.3.4 Adaptor proteins

Grb10 and Grb14, as well as proteins of the suppressor of cytokine signaling (SOCS) family (especially SOCS1 and SOCS3), are negative regulators of the tyrosine kinase activity of the IR, proving that it has a role in insulating the contact of IR substrates to the activated receptors. (34)

Tribbles homolog 3 (Trb3) pertains to a family of pseudokinases that could bind to AKT and block its activation, hence inhibiting insulin signaling. Besides, inositol phosphate IP7 could inhibit AKT translocation to the cell surface, thus attenuating insulin signaling (34).

## 3 Insulin resistance mechanism

Insulin resistance is typical with a state when plasma insulin is at a normal range; however, target tissues cannot exert a physiological response of glucose-lowering, implicating the inhibition of gluconeogenesis, lipolysis, cellular glucose uptake, and glyconeogenesis (2, 41). The state of insulin resistance commands enhanced insulin secretion to recompense; hence, fasting plasma insulin is in high concentrations (42). In this review, insulin resistance is defined as a curve of responding to insulin dose with increased EC_50_ (half maximal effective concentration), with or without declined maximal response (43).

The physiological mechanism of insulin resistance owes to deficient insulin action at target cells. To pinpoint the deficiency of cellular insulin action, a lot of effort has been induced and two major origins have been indicated: receptor defects (decreased IR expression at the plasma membrane) and post-receptor defects (impaired signal transduction) (44). In the case of decreased IR expression (receptor defects), the dose response increased but the maximal biological response remained the same at the normal value unless the IR fell to 5-10% of the normal value. Distinctly, not only was there an increase in dose response but the maximal response also decreased in the case of post-receptor defects (2, 44, 45).

Receptor defects lead to insulin resistance by mutations of IR in the majority. Mutations of IR exert the type A insulin resistance syndrome, which occurs in at least 0.05% of the general Japanese population (46, 47). Apart from that, type B insulin resistance syndrome causes insulin resistance by autoantibodies blocking the binding sites of insulin on IR. This syndrome is frequently accompanied by autoimmune conditions (48). Besides, Rabson–Mendenhall syndrome and Donohue syndrome are also brought about by mutations of IR, most of which are in both alleles but are more serious compared to type A insulin resistance syndrome, which is usually associated with intractable diabetes (47, 49).

Post-receptor defects comprise abnormalities in metabolic signals and mitogenic signals; it includes the effects on substrates and negative modulators of insulin signaling.

### 3.1 Substrates of insulin signaling

Many single nucleotide polymorphisms (SNPs) and gene mutations of IRS have been identified that are associated with decreased insulin signaling. The SNPs G972R, Gly972Arg, and rs1801278 of IRS-1 have shown a high prevalence in patients with type 2 diabetes and are caused by disrupted insulin signaling (50–52). It showed that the T608R mutation was located in the highly conversed region of IRS-1 that impairs PI 3-kinase activation and binding, as well as GLUT4 translocation in adipose cells. On the other hand, the I65S, R66S, and G86R mutations reflexed amino acids in the phosphotyrosine binding site of the IRS-1 protein. These changes in amino acids could lead to a shift in binding energies and trigger conformational variations in the L1 domain of the IR structure. Besides, in the ligand-binding area of the IR structure, I65S, R66S, and G86R mutations of IRS1 structures’ positional displacement were caused by the observation of variable binding mode orientations. All of these factors could be reasons for the varied interactions of IRS1 and IR, which lead to aberrant insulin transduction, followed by insulin resistance (53, 54).

The activity of PI3K depends on an equivalence between the activities of two subunits: the regulatory and catalytic subunits (55). In the regulatory subunit, several mutations have shown their association with signal transduction. The mutations of PIK3R1, encoding the p85α regulatory subunit of PI3K, including the most frequent mutation Arg649Trp and 11 other PIK3R1 mutations have been recognized in patients with SHORT syndrome and genetic insulin resistance. The majority of the mutations in this syndrome are in the area encoding the C-terminal SH2 domain of p85, which is required for PI3K binding to tyrosine-phosphorylated proteins including insulin receptor and (IR) substrate (IRS-1). The 649 amino acid position on the SH2 domain of the p85 regulatory subunit is arginine (wild-type) and highly conserved, and it participates in a bivalent interaction between PI3K and IR or IRS with the linkage of oxygen atoms of the phosphotyrosine. The improved link to the phosphopeptide was focused within the binding pocket. The mutation Arg649Trp at this location changes the amino acid sequence from Arginine to Tryptophan, which decreases affinity to the phosphopeptide (49). Several works of research have indicated that PIK3R1 mutations are the second common origin in the rank of single-gene insulin resistance (56–58). Mutations of PIK3R2, encoding the p85β regulatory subunit of PI3K, have shown the ability to augment PI3K signaling, which has been perceived in patients with hypoglycemia and either segmental overgrowth or megalencephaly (59). Somatic mutations of PIK3CA, encoding the p110α catalytic subunit of PI3K have been seen generally in tumor cells and segmental overgrowth tissues less frequently, while germline mutations of PIK3CA have been observed in patients with segmental overgrowth or megalencephaly (59, 60).

AKT substrate includes three isoforms: AKT1, AKT2, and AKT3; wherein AKT1 and AKT2 are universally occurring, AKT3 is mainly expressed in the central nervous system (2). The mutations of AKT1 and AKT3 have not shown an association with insulin sensitivity; however, in contrast, mutations of AKT2 are an identified origin of the monogenic disorder of glucose metabolism (61). A heterozygous Arg274His mutation of AKT2 gives rise to a blocked impact on insulin activity, which was observed in patients with severe postprandial hyperinsulinemia (62). A lower function AKT2 coding variant, p.Pro50Thr, occurs with high frequency (1.1%) in the Finnish population, and influences glucose uptake negotiated by insulin in target tissues, thus escalating the risk of type 2 diabetes in carriers (23, 63).

TBC1D4 is involved in GSV translocation, and its mutations may affect insulin action. A truncated Arg363Ter mutation was identified in a family who suffered from postprandial hyperinsulinemia (64). A homozygous Arg684Ter variant of TBC1D4, which appeared with a great allele prevalence (17%) in the Greenlandic population, has shown a remarkable increase in the risk of the progress of type 2 diabetes (65). Deletion of TBC1D4 generating abolished glucose uptake negotiated by insulin is related to impaired glycemic control (66).

The variants in PTPN11 are possibly responsible for the blockage of tyrosine kinase signaling and downstream Ras-MAPK signaling, hence exerting Noonan syndrome. The individuals harboring mutations of PTPN11 manifest insulin resistance with impairment in glucose uptake and glycogen synthesis (67, 68).

### 3.2 Negative modulators of insulin signaling

Patients with Cowden syndrome are heterozygous mutations in PTEN for diminished function and also have elevated insulin sensitivity. PTEN haploinsufficiency and PTEN deletion ameliorate insulin sensitivity and defend against systemic insulin resistance associated with obesity. Besides, the inactivation of PTEN gives rise to the decreased hyperproliferation of cells (69, 70).

Several factors could exert increased activity of stress kinases including JNK, IKK, and PKC, thus increasing insulin resistance state. Some of these factors include free fatty acids (FFA), diacylglycerol (DAG), sphingolipid ceramide, hyperglycemia, reactive oxygen species (ROS), and endoplasmic reticulum (ER) stress (34).

Obesity has an association with insulin resistance, which is characterized by the progress of chronic inflammation at a low level (71). Adipocytes secreting the chemokine MCP-1 promote macrophage agglomeration into adipose tissues and exert insulin resistance (72). Several cytokines, such as TNF-α, IL1β, and IL-6, are secreted by immune cells and adipocytes that give rise to insulin resistance by increased serine/threonine phosphorylation or activation of SOCS3 (an adaptor protein) in adipocytes (73, 74).

Interestingly, in the context of insulin resistance, when the metabolic signals such as PI3K signaling is inhibited, the mitogenic signals like the Ras-Raf-MAPK pathway of insulin are not interrupted and are possibly upregulated (75).

## 4 Applications

Insulin resistance leads to increased insulin secretion to compensate; this state is known as hyperinsulinemia (42). Insulin resistance and/or hyperinsulinemia could exert many complications, such as obesity, cardiovascular disease, neurodegeneration especially Alzheimer’s disease, and cancer (3).

Indeed, insulin resistance could result in lipoproteins’ profile alterations. ApoB is a protein that could enhance the assemblage and excretion of VLDL. Normally, through the PI3K pathway, insulin controls the deterioration of apoB; however, under insulin resistance, this process is inadequate. Besides, insulin resistance also decreases the activity of lipoprotein lipase, which is a dominant factor in VLDL clearance. Thereby, insulin resistance gives rise to an escalation in VLDL formation, which accounts for hypertriglyceridemia under the insulin resistance state (76–79). Insulin resistance also exerts the synthesis of small dense LDL (sdLDL) and decreases HDL levels, which are created by the transferring of VLDL’s triglycerides to LDL and HDL under the catalysis of cholesteryl ester transfer protein (CETP), provoking triglyceride-rich LDL and decreasing HDL-C. Triglyceride-rich LDL is lipolysis by hepatic lipase, leading to the formation of sdLDL. sdLDL has several properties including lessened affinity for the LDL receptor, accelerated entry into the arterial wall, dominant arterial detention, major sensitivity to oxidation, and greater half-life that promotes the atherogenic activity of sdLDL. Strikingly, increased sdLDL levels could not be measured by the LDL levels, due to the lower level of sdLDL than LDL. These variations of lipoproteins profile are called dyslipidemia, which is characterized by the lipid trinity: hypertriglyceridemia, low levels of HDL, and the occurrence of sdLDL, which leads to obesity. Dyslipidemia is a sturdy predictor of the development of type 2 diabetes, which is owed to insulin resistance and cardiovascular disease (9, 80–82). A strong association between insulin resistance and the risk of cardiovascular disease (CVD) has been indicated (83). Several molecular mechanisms contributing to this correlation include atherosclerosis development and vascular function (9, 84). Dyslipidemia is the main reason for atheroma plaque formation. Besides, insulin resistance could exert vascular abnormality by activating the MAPK pathway generating vasoconstriction improvement and inhibiting the NO synthesis which has an essential role in vascular strength through the ability of vasodilation (85).

Several studies have revealed that brain insulin resistance, which means dysregulated brain insulin signaling, is one of the factors of cognitive disorders and Alzheimer’s disease (AD) and is also considered a risk factor for sporadic AD development (86, 87). Indeed, the major pathology of AD including neurofibrillary tangles (NFT) and amyloid-β plaques could owe to insulin resistance (8). Firstly, insulin, IGF-1, and IGF-2 have been indicated to have inhibited activity on apoptosis in brain neurons through IR and regulate many neurobiological processes, inhibiting the degradation of amyloid-β (88–90). Moreover, amyloid-β could compete with insulin for binding to IR, leading to impairment of insulin-binding affinity to IR, and thus exerting insulin resistance. This state has been proven to aggravate the already present AD pathology (91). The increased serine phosphorylation of IRS-1, PI3K pathway dysfunction, and inhibition of AKT causing stimulation of Glycogen synthase kinase 3β (GSK-3β) and mTOR phosphorylation lead to hyperphosphorylation of tau protein, which is the main component of NFT. Besides, activated GSK-3β could enhance the amyloid-β plaque deposition. Both of them may give rise to microglia-mediated neuroinflammation that produces AD pathogenesis (92–96). Apart from that, the activation of the Ras-MAPK pathway enhances the transcription of several particular genes related to the role of the survival of neurons which are associated with synaptic plasticity and tau phosphorylation and are connected with amyloid-β plaques accumulation and NFT formation, which are major features of AD (97, 98).

Several investigations have indicated the association between hyperinsulinemia with the risk of certain types of cancers. This phenomenon could be analyzed by direct or indirect impacts of insulin resistance on the development of cancer (3). The direct impacts due to the overexpression of insulin resistance in specific tumor cell lines enhance the proliferative response to insulin, and ERK has been pointed out to be responsible for activating the cell cycle process in cancer cells (99, 100). The indirect effects account for the ability of insulin to stimulate increased levels of many modulators of proliferation and differentiation including IGF-1, cytokines, and growth factors, such as leptin, VEGF, and IL-6 (101–103). These effects may accelerate the growth of neoplasms, angiogenic process, and metastasis, and could be the mechanisms by which hyperinsulinemia and/or insulin resistance increases the carcinogenesis risk of many kinds of cancer, such as cancer of the breast, colon, liver, pancreas, and endometrium (104–107).

Based on these observations, it would seem that IR modulator therapies are effective in the treatment of diseases related to IR, especially for those substances that more preferentially stimulate metabolic signaling (AKT), minimally activate mitogenic responses (ERK), and limit insulin-like growth factor receptor (IGFR) activation (3, 108). Several reports have declared some IR agonist molecules that would be satisfied to become a safe and efficient IR agonist (selectively activating the AKT pathway over the ERK signaling): the peptide S597 and the human monoclonal antibody XMetA (3, 109, 110).

## 5 Conclusion

The complications of insulin signaling do not only owe to the requirement for maintaining the plasma glucose within a narrow range and the regulation of other metabolic substances but also control the growth and survival of multiple cells. The ability to design IR agonists provides variable potencies for treatment. While extensive investigations have been made, a lot of gaps persist in our knowledge of the molecular mechanisms involved in insulin signaling and will stand as the highlight of years of studies. It is hoped that this review would serve as a resource for assistance in the progress of more efficient next-generation therapies for diseases related to insulin resistance.

## Author contributions

CL and TN have equally contributed as the main authors to the supervision of the final manuscript. All authors contributed to the article and approved the submitted version.
